# RocTest: A standardized method to assess the performance of root organ cultures in the propagation of arbuscular mycorrhizal fungi

**DOI:** 10.3389/fmicb.2022.937912

**Published:** 2022-07-28

**Authors:** Dane Goh, Julien G. A. Martin, Claudia Banchini, Allyson M. MacLean, Franck Stefani

**Affiliations:** ^1^Department of Biology, University of Ottawa, Ottawa, ON, Canada; ^2^Agriculture and Agri-Food Canada, Ottawa Research and Development Centre, Ottawa, ON, Canada

**Keywords:** Medicago, modeling, mycorrhizal, Nicotiana, root, spore, symbiosis

## Abstract

Over the past three decades, root organ cultures (ROCs) have been the gold standard method for studying arbuscular mycorrhizal fungi (AMF) under *in vitro* conditions, and ROCs derived from various plant species have been used as hosts for AM monoxenic cultures. While there is compelling evidence that host identity can significantly modify AMF fitness, there is currently no standardized methodology to assess the performance of ROCs in the propagation of their fungal symbionts. We describe *RocTest*, a robust methodological approach that models the propagation of AMF in symbiosis with ROCs. The development of extraradical fungal structures and the pattern of sporulation are modeled using cumulative link mixed models and linear mixed models. We demonstrate functionality of *RocTest* by evaluating the performance of three species of ROCs (*Daucus carota*, *Medicago truncatula*, *Nicotiana benthamiana)* in the propagation of three species of AMF (*Rhizophagus clarus*, *Rhizophagus irregularis*, *Glomus* sp.). *RocTest* produces a simple graphical output to assess the performance of ROCs and shows that fungal propagation depends on the three-way interaction between ROC, AMF, and time. *RocTest* makes it possible to identify the best combination of host/AMF for fungal development and spore production, making it an important asset for germplasm collections and AMF research.

## Introduction

Arbuscular mycorrhizal fungi (AMF) are obligate biotrophs, which colonize the roots of diverse plants to establish symbioses with 72–80% of terrestrial species (Brundrett and Tedersoo, 2018). Much knowledge about these soil fungal symbionts comes from *in vitro* mycorrhiza established on root organ cultures (ROCs). These genetically transformed roots (“hairy roots”), obtained by the insertion of the Ri T-DNA plasmid from *Rhizobium rhizogenes* into plant tissues, grow quickly and continuously. Despite a modified hormonal balance (Fortin et al., 2002), they are morphologically, physiologically, and metabolically similar to their autotrophic counterparts (Bago and Cano, 2005), and AMF can colonize transformed roots. This culturing method provides highly controlled conditions and enables real-time, non-destructive monitoring of the extraradical development of the fungal symbiont (Bago et al., 1998; Declerck et al., 2001; Fortin et al., 2002).

The development of extraradical fungal structures reflects the performance of hairy root hosts in propagating AMF in monoxenic cultures. Indeed, AMF receive sugars and fatty acids from their host (whether the host being an entire plant or a Ri T-DNA root), which are then stored in spores (Bago et al., 2003; Bravo et al., 2017; Luginbuehl et al., 2017; Rich et al., 2017). However, different approaches and experimental procedures are frequently applied to monitor the extraradical development of the fungal symbiont, making it difficult to compare the performance of hairy roots as hosts across studies. Hairy roots from various plant species have been trialed to cultivate AMF *in vitro* (i.e., Bago and Cano, 2005; Puri and Adholeya, 2013). Among these, carrot (*Daucus carota*) roots are commonly used as a root organ for AM monoxenic cultures (Cranenbrouck et al., 2005; Kokkoris and Hart, 2019). Carrot roots show a consistent and vigourous growth (Bécard and Fortin, 1988) and support an abundant production of spores when inoculated with some *Rhizophagus* species (St-Arnaud et al., 1996; Douds, 2002; Rosikiewicz et al., 2017). Root organs from other plant species are also used to propagate AMF, such as *Cichorium intybus* (Verdin et al., 2006), *Glycine max* (Fernandez et al., 2009), *Linum usitatissimum* (Rodrigues and Rodrigues, 2015), *Lycopersicon esculentum* (Simoneau et al., 1994), *Medicago truncatula* (Boisson-Dernier et al., 2001) or *Solanum tuberosum* (Angelard et al., 2014). There is currently no tool to assess their performance against each other or against the carrot root organs commonly used in germplasms. Characterizing and comparing the ability of hairy roots to propagate AMF requires a standardized methodological approach.

A standardized framework is also necessary to screen for the best host/fungus combination. The identification of the best pairing is a reasonable goal toward increasing the diversity and stability of AM monoxenic cultures. About 20 AMF species have been established on ROCs (Rodrigues and Rodrigues, 2015), but their propagation over multiple generations is not guaranteed, as the fungi can stop growing after a few subcultures. Thus, fewer than 10 of 343 known AM species^1^ are perpetually maintained under *in vitro* conditions in international collections (Mycothèque de L’UCLouvain-Belgian Co-ordinated Collections of Microorganisms – BCCM/MUCL^2^, Canadian Collection of AMF – CCAMF^3^.

The impact of host diversity on the propagation of AMF is underexplored, although there is compelling evidence that host identity can modify AMF fitness. Within ecosystems, AMF exhibit a non-random distribution amongst different host species (Eom et al., 2000; Croll et al., 2008; Davison et al., 2011), suggesting preferences for different plant species. Within Petri dishes, different clones or species of root organs inoculated with the same AM fungal strain can modify the fungal phenotypes (hyphal and spore density) and genotypes (Tiwari and Adholeya, 2003; Ehinger et al., 2009; Angelard et al., 2014). For instance, the ratios of nucleotypes in spores from the heterokaryotic strains of *R. irregularis* vary according to the host identity (Kokkoris et al., 2021). The host and fungal genotypes can alter the transcriptional responses of each partner following the symbiosis (Mateus et al., 2019). Therefore, fungal fitness is intimately linked to host identity, and successful AM monoxenic cultivation requires assessment of a greater diversity of AM species over time in combination with a greater diversity of host roots.

Here we introduce *RocTest*, a standardized framework to evaluate the performance of root organs as hosts to AMF and to visualize the best pairing among a set of ROCs and AMF. *RocTest* models the ROC performance using stacked probabilities inferred from the development of the fungal symbiont and investigates the three-way interaction between host, AMF, and time. It can also model the host impact on the pattern of sporulation. *RocTest* modeling of one-to-one pairings of host root and AMF relies on cumulative link mixed models and general linear mixed models. We demonstrate the functionality of *RocTest* by evaluating the performance of ROCs derived from three host species (*D. carota*, *Medicago truncatula*, and *Nicotiana benthamiana)* in the propagation of AM fungal species (*Rhizophagus clarus*, *R. irregularis*, and *Glomus* sp.). The *RocTest* scripts and instructions on how to easily implement *RocTest* in any laboratory are available from the open science framework (OSF) repository.

## Methods

Monoxenic cultures were set up using three species of root organs and three AM fungal species. Root organs (Supplementary Figure 1) were derived from *Nicotiana benthamiana* (hereafter Nicotiana), *Medicago truncatula* (ecotype R108; hereafter Medicago), and *D. carota* (clone P68; hereafter Daucus), as described further in Supporting Information. Fungal inocula of *Rhizophagus irregularis* (DAOMC234181), *Rhizophagus clarus* (DAOMC234281), and *Glomus* sp. (DAOMC240160), were provided by the Canadian Collection of Arbuscular Mycorrhizal Fungi (CCAMF; Agriculture and Agri-Food Canada, Ottawa, ON, Canada, Supplementary Table 1). *Glomus* sp. (DAOMC240160) is an undescribed species related to the genus *Rhizophagus*. All fungal species were previously maintained under monoxenic conditions in association with Ri T-DNA transformed *D. carota* on modified Strullu-Romand medium (Diop, 1995; Declerck et al., 1996). The host impact on AMF propagation was assessed and compared using a factorial design with three levels of host (Nicotiana, Medicago, and Daucus) and AMF (*R. irregularis*, *R. clarus*, and *Glomus* sp.) This resulted in nine pairs of host/AM fungal species. More than 50 monoxenic cultures are performed for each pair in order to have enough cultures to analyze until the 12th week of the experiment (Supplementary Table 2).

### Preparation of *in vitro* cultures

**FIGURE 1:**
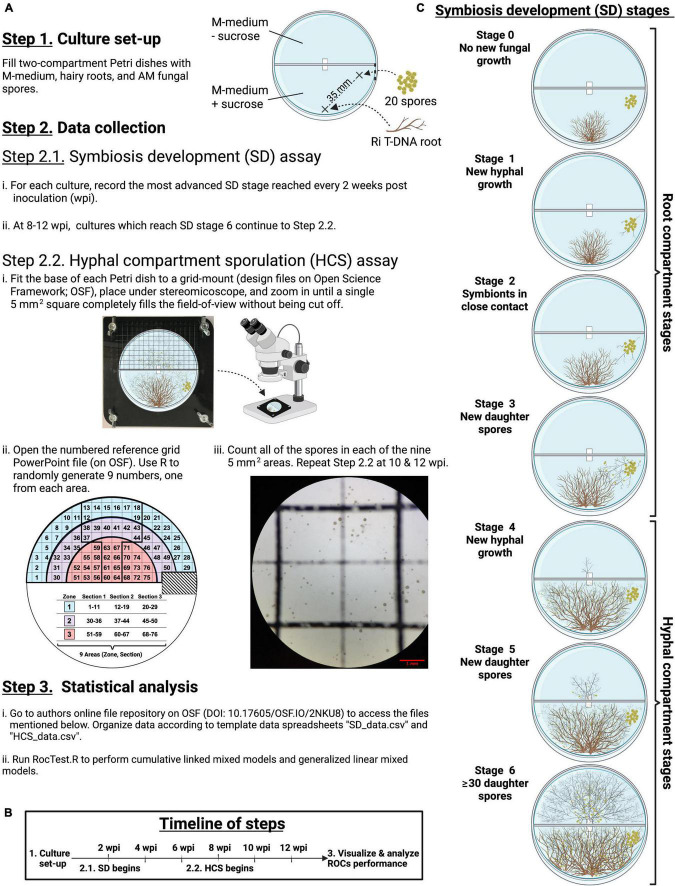
Step-by-step flowchart of the experimental design **(A)**, the timeline **(B)**, and illustrations of each stage of the symbiosis development **(C)**.

Monoxenic cultures were set up using two-compartment Petri dishes (Corning™ or Kord Valmark™, 100 mm × 15 mm, Figure 1A, step 1). The fungal propagation was monitored in each root compartment (RC) and each hyphal compartment (HC; free of roots). Compartments were filled with minimal (M) medium (Bécard and Fortin, 1988), modified as follows: pH was adjusted to 6.0, phytagel (Sigma-Aldrich) was used as a gelling agent (3 g/l), and the M medium in each RC was complemented with 20 g/l of sucrose according to St-Arnaud et al. (1996). An autoclaved filter paper bridge (1 cm × 3 cm) was placed over the center of the plastic barrier to facilitate fungal propagation from a RC to a HC (Dalpé and Séguin, 2010). Each tip of the paper bridge was partly embedded into the medium. The root sample and the spores were consistently positioned at the same place in the RC across all replicates (Figure 1A, Step 1). For each trial, the RC of all cultures were inoculated with 20 spores from the same *in vitro* “mother” culture. The culture medium of the *in vitro* “mother” culture was solubilized using a citrate buffer (Doner and Bécard, 1991) to release the spores. Inoculation of spores took place under a flow hood, where spores were aseptically manipulated using a stereomicroscope and syringe needles.

### Data for running *RocTest*

The extraradical development of the fungal symbiont in each RC and HC (hereafter symbiosis development – SD) was monitored every 2 weeks during 3 months to model the ROC performance (Figures 1A–C, Steps 2.1). Seven mutually exclusive sequential stages (0 to 6) were scored to assess the SD (Figure 1C). Stages 0 to 3 occurred in the RC, whereas stages 4 to 6 took place in the HC. Stage 0 represented the time between the culture set-up and the production of new hyphae. Stage 1 was defined as the production of new hyphae growing away from the initial inoculum of 20 spores. Stage 2 was scored when roots and hyphae came into close contact (≤1 mm) with one another. Stage 3 was scored at the first observation of new spore(s) in the RC. Stage 4 represented the stage where hyphae crossed the paper bridge into the HC. Stage 5 corresponded to the production of new daughter spores in HC. Lastly, stage 6 was scored when at least 30 spores were counted in the HC, in accordance with the typical bacteria colony-forming units (CFUs) plate counting method (Breed and Dotterrer, 1916). Each of the SD stages are distinct from one another and are therefore easily distinguishable under a stereomicroscope.

The number of spores produced in each HC, along with their distance to the hyphal bridge, were monitored twice a week to model the host impact on the pattern of sporulation (Figure 1A, Steps 2.2). This was estimated using cultures that had reached stage 6 by 8, 10, or 12 weeks post inoculation (wpi, Figure 1C). The HC was divided into squares of 5 mm^2^ using a laser-cut grid-mount (design files on OSF, DOI 10.17605/OSF.IO/2NKU8). The grid-mount made it possible to overlay the grid, printed on projector paper, on top of the HC (inverted position) and to lock all components into a consistent position for all observations (Figure 1A, Step 2.2 i). Then, three semicircular zones were defined in the HC based on the distance between the squares and the hyphal bridge: zone 3 included squares at a radial distance of 0–20 mm from the bridge, while zones 2 and 1 included squares at a radial distance of 21–30 mm and 31–40 mm from the bridge, respectively (Figure 1A, Step 2.2 ii). Although some squares fell across two zones, a square was considered to belong to a particular zone if most of its surface (∼80%) was within that zone. A total of 76 squares were distributed across the three zones. Each zone was then sub-divided into three areas each with an equal number of squares in order to evenly distribute three randomly selected squares per zone. Observation of these nine randomly selected squares provided a representative snapshot of sporulation inside the HC. Spores were counted in each selected square at 32 × magnification using a stereomicroscope (Zeiss Discovery V8) with CL 1500 ECO as a light source (Figure 1A, Step 2 iii). A unique set of nine numbers was generated for each bi-weekly observation of each Petri dish for all pairs of host/AMF.

The distance between each of the 76 squares and the hyphal bridge was determined as follows: first, the dimensions of a two-compartment Petri dish (100 mm × 15 mm) were measured to create a reference image on PowerPoint (PowerPoint file on OSF). Next, the reference image was imported to FIJI v2.1.0/1.53c (Schindelin et al., 2012) to measure the distance between each square and the bridge.

### Statistical analyses and modeling methods

To model host performance in the propagation of AMF, the successive SD stages (0 to 6) were considered an ordinal variable and analyzed using cumulative link mixed models (Figure 1A, Step 3 ii). The probability of reaching a given stage was modeled as a function of host, AMF, and time; this three-way interaction made it possible to fit varying developmental rates specific to each combination of host/AMF. To control for repeated measurements within the same Petri dish over multiple time points, cultures were assigned unique IDs (Petri dish number, trial number, host, and AMF). The model was fitted using the *clmm* function from the ordinal package (Christensen, 2019). The probability associated with each effect was estimated using likelihood ratio tests with the *Anova.clmm* function from the RVAideMemoire package (Hervé, 2021). Note that testing on single terms when a three-way interaction is included in the model may not be powerful enough and results in a high probability estimate (see Table 1). Finally, a *post-hoc* pairwise comparison was performed to determine if the progression rate of specific host/AMF pairs through the SD stages differed from each other.

**TABLE 1:** Cumulative link mixed models comparing the progression of four pairs of host (Medicago, Daucus)/AMF (*R. irregularis, Glomus* sp.) through the six stages of symbiosis development as a function of a three-way interaction between host, AMF, and time (0–12 weeks).

Coefficients	Estimate	SE	Chi square	Df	*p*-value
**AMF**	–	–	<0.001	2.00	>0.99
*Glomus* sp.	1.79	0.78	–	–	–
*R. irregularis*	2.49	0.72	–	–	–
**Host**	–	–	<0.001	2.00	>0.99
Daucus	1.47	0.75	–	–	–
Medicago	2.56	0.74	–	–	–
**Time**	0.20	0.06	<0.001	1.00	>0.99
**AMF × Host**	–	–	12.28	4.00	0.015
*Glomus* sp. × Daucus	−3.04	1.09	–	–	–
*R. irregularis* × Daucus	−2.12	0.98	–	–	–
*Glomus* sp. × Medicago	−1.94	1.05	–	–	–
*R. irregularis* × Medicago	−2.66	0.98	–	–	–
**AMF × Time**	–	–	<0.001	2.00	>0.99
*Glomus* sp. × Time	0.21	0.08	–	–	–
*R. irregularis* × Time	0.09	0.08	–	–	–
**Time × Host**	–	–	<0.001	2.00	1
Time × Daucus	0.73	0.08	–	–	–
Time × Medicago	0.29	0.08	–	–	–
**AMF × Host × Time**	–	–	54.56	4.00	<0.001
*Glomus* sp. × Time × Daucus	−0.56	0.11	–	–	–
*R. irregularis* × Time × Daucus	−0.07	0.11	–	–	–
*Glomus* sp. × Time × Medicago	−0.30	0.11	–	–	–
*R. irregularis* × Time × Medicago	0.32	0.11	–	–	–

Estimates and standard error (SE) were obtained from MLR. Chi square values, degrees of freedom (Df), and *p*-value were obtained from type III ANOVA.

To test the host effect on the pattern of sporulation (distance to the hyphal bridge and number of spores), a generalized linear mixed model (GLMM, Figure 1A, Step 3 iii) of the number of spores per square was fitted with a Poisson error distribution using the *glmer* function from the lme4 package (Bates et al., 2015). Host, AMF, time, and their three-way interaction were fitted as fixed effects. Furthermore, it was predicted that as time progressed, sporulation would occur farther into the HC. This represented a greater distance from the hyphal bridge (i.e., the hyphae’s initial point of contact with the HC); therefore, the interaction between time and distance from the bridge was also included as a fixed effect for sporulation. Repeated measurements were controlled as explained in the above paragraph. Data from the cultures inoculated with *Glomus* sp. were not included in the model because not enough cultures produced spores in the HC with Daucus and Nicotiana as host (Supplementary Table 3). The sample size is also limited for *R. irregularis* and *R. clarus* with Nicotiana as a host (9 squares, only one Petri dish). This limited sample size explains the wide confidence intervals estimated on Nicotiana in this analysis. Removing all samples taken on Nicotiana provide qualitatively similar results. Data collected on Nicotiana were used in the analysis to illustrate the statistical approach but estimates for Nicotiana should be interpreted with care.

The GLMM was followed by Type III ANOVAs to estimate the probability associated with the fixed effect estimates. Each ANOVA was followed by a Tukey HSD *post-hoc* test for pairwise comparisons between unique pairs of host/AMF (Supplementary Table 4). The type III ANOVA tests if overall there are differences in spore production across all host AMF combinations and the *post-hoc* tests allow to statistically compare the spore production between pairwise combination of AMF and host.

All statistical analyses were performed using the R software v4.0.3 (R Core Team, 2020). Instructions, input files, and the script *RocTest.R* are available on the OSF project by Goh et al. (2022)^4^ (Figure 1A, Step 3 i).

## Results

### Profiling root organ culture performance with stacked probabilities of symbiosis development

**FIGURE 2:**
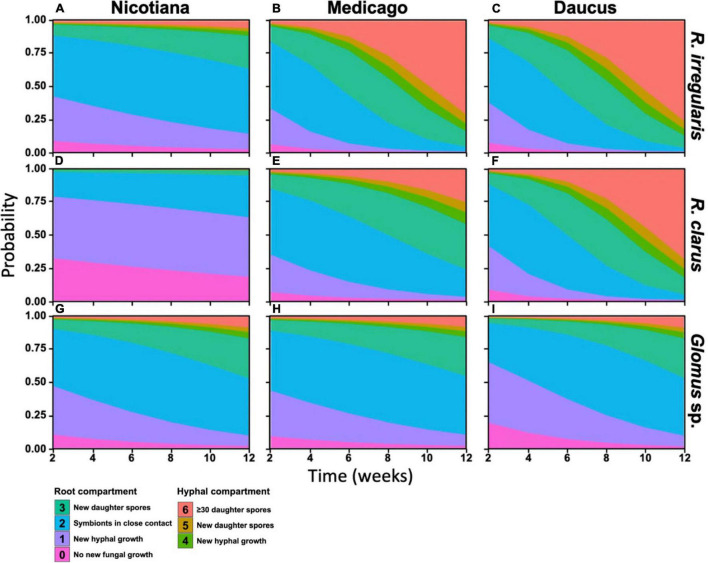
Stacked probabilities of each pair of host and AM fungal species. Distribution of colored polygons correspond to the probability distribution of any pair host and AM fungus replicate reaching a developmental stage (see Figure 1C) at any given time during the 12-week assay period. Probabilities were generated by testing fungal development as a function of a three-way interaction between host identity, fungal identity, and time (in weeks) using a multinomial linear regression.

*RocTest* produces a simple graphical output to assess the performance of hairy roots in the propagation of AMF in monoxenic cultures (Figure 2). It also shows that the factors influencing fungal propagation are more complex than the host effect alone. Indeed, fungal propagation depended on the three-way interaction between host, AMF, and time (*p* < 0.001, Table 1). The different combinations of host/AMF were easy to analyze using the stacked probability plots and the statistical analyses included in *RocTest*. Thus, Nicotiana was identified as a poor host for the three fungal species (Figures 2A,D,G), and *Glomus* sp., as a difficult species to propagate with the three root organs (Figures 2G–I).

*RocTest* also analyses the variability in propagation between two pairs of host/AMF. For instance, the progression of *R. irregularis* through the SD stages was significantly slower when in symbiosis with Nicotiana than with Medicago (χ^2^_1_ = 46.5, *p* < 0.001) and Daucus (χ^2^_1_ = 68.03, *p* < 0.001) (Figures 2A–C). The probability of producing new daughter spores in the RC (stage 3) at 12 wpi was 29% for *R. irregularis* in symbiosis with Nicotiana, whereas it was 99% when in symbiosis with Medicago and Daucus. Further similarity in *R. irregularis* development between Medicago- and Daucus-hosted cultures was observed when comparing the fungal growth in the HC (stages 4 to 6). Throughout the 12 weeks of incubation, the probability of *R. irregularis* producing more than 30 spores in the HC (stage 6) doubled every week in both Medicago and Daucus cultures. Taken together, *R. irregularis* progressed through the SD stages at a similar pace (Figures 2B,C) when hosted by Medicago or Daucus (χ^2^_1_ = 0.61, *p* = 0.43).

With respect to the propagation of *R. clarus* (Figures 2D–F), Nicotiana was the weakest host in comparison to Medicago (χ^2^_1_ = 17.36, *p* < 0.001) and Daucus (χ^2^_1_ = 88.7, *p* < 0.001), while Daucus was a better host than Medicago (χ^2^_1_ = 55.97, *p* < 0.001). The probability of reaching stages 4 or above at 12 wpi was 25% for *R. clarus* in symbiosis with Medicago, whereas it was 96% when in symbiosis with Daucus. The probability of specifically reaching stage 6 at 12 wpi was 86% for *R. clarus* in symbiosis with Daucus, compared to 8.4% when in symbiosis with Medicago. Finally, *Glomus* sp. was not productive with any of the three species of ROCs (Figures 2G–I). Notwithstanding, a significant difference was found between Medicago and Daucus (χ^2^_1_ = 17, *p* < 0.001), indicating that *Glomus* sp. progressed through the SD stages faster with Daucus in comparison to Medicago.

### Host effect on sporulation

**FIGURE 3:**
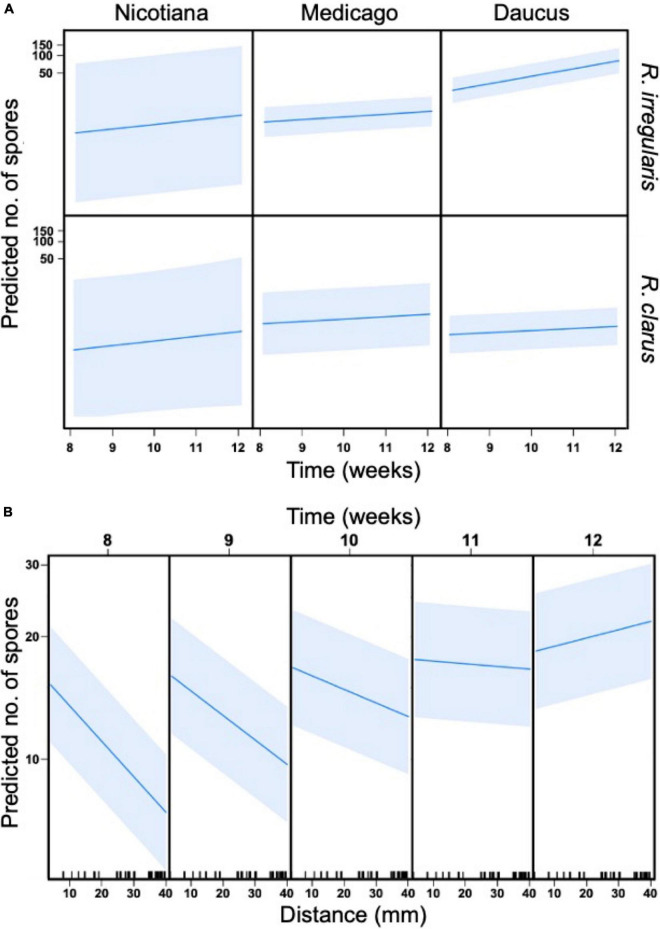
General linear mixed models (GLMM) with four predictor variables, host (Nicotiana, Medicago, Daucus), AMF (*R. irregularis* and *R. clarus*), time (8, 10, and 12 wpi), and distance (mm, location of grid squares in relation to hyphal bridge), and their effect on spore production (dependent variable). Petri dish ID (individual replicates) were used as random effect. **(A)** Three-way interaction between time, AMF, and host. The slope of each individual graph indicates a relationship between predictors and spore production, while the shaded area illustrates standard error. **(B)** Two-way interaction between distance and time.

The pattern of sporulation in the HC was also dependent on the three-way interaction between host, AMF, and time (*p* < 0.001, Figure 3A and Table 2). The host identity affected the number of spores recorded in the hyphal compartment for *R. irregularis* only (Supplementary Table 4). From weeks 8 to 12, *R. irregularis* produced more spores in the HC when it was propagated with Daucus than with Medicago (*p* < 0.001). For instance, the average number of spores counted in each square of a plate at 12 wpi was 6.2 times higher with Daucus ($\bar{x}$ = 125) than with Medicago ($\bar{x}$ = 20). This difference was even more pronounced between Daucus and Nicotiana ($\bar{x}$ = 9.2). However, the difference was not significant (*p* = 0.73), likely due to the limited number of cultures with Nicotiana that reached week 12 (many of the cultures were lost due to contamination). Despite the earlier differences in SD stage progression, *R. clarus* sporulation was similar in rate as well as spatial distribution, regardless of host species. For instance, the average number of spores counted in each square of a plate by week 12 was similar (*p* = 0.99) between Daucus ($\bar{x}$ = 6.3) and Medicago ($\bar{x}$ = 10). Since too few cultures inoculated with *Glomus* sp. reached the sixth SD stage, no comparison could be made with respect to the effect of host performance on sporulation into the HC.

**TABLE 2:** The general linear mixed model (GLMM) comparing the sporulation at 8–12 wpi in the hyphal compartment (HC) of four pairs of host (Medicago and Daucus) and AMF (*R. irregularis* and *R. clarus*) as a function of a three-way interaction between host, AMF, and time (weeks).

Fixed effects	Estimate	Std. error	Chi square	Df	*p*-value
**(intercept)**	0.65	2.17	0.09	1.00	0.77
**Distance**	−0.07	0.00	624.07	1.00	**<0.001**
**Time**	0.02	0.18	0.02	1.00	0.90
**AMF**	–	–	0.24	1.00	0.62
*R. irregularis*	1.30	2.62	–	–	–
**Host**	–	–	0.64	2.00	0.73
Daucus	1.43	2.21	–	–	–
Medicago	1.74	2.27	–	–	–
**Distance × Time**	0.01	0.00	578.02	1.00	**<0.001**
**AMF × Host**	–	–	0.07	2.00	0.96
*R. irregularis* × Daucus	−0.65	2.67	–	–	–
*R. irregularis* × Medicago	−0.73	2.72	–	–	–
**Time × AMF**	–	–	0.00	1.00	0.98
Time × *R. irregularis*	−0.01	0.19	–	–	–
**Time × Host**	–	–	0.48	2.00	0.79
Time × Daucus	−0.10	0.18	–	–	–
Time × Medicago	−0.09	0.18	–	–	–
**Time × AMF × Host**	–	–	51.18	2.00	**<0.001**
Time × *R. irregularis* × Daucus	0.22	0.19	–	–	–
Time × *R. irregularis* × Medicago	0.02	0.19	–	–	–

Intercept refers to the sporulation at 8 wpi in the HC of the pair Nicotiana/R. clarus. Estimate and standard error (Std. Error) obtained from IC model. Chi square values, degrees of freedom (Df), and *p*-values were obtained from type III ANOVA.

Sporulation in the hyphal compartment (HC) depended on the distance to the hyphal bridge. The number of spores recorded in any given square in the HC decreased significantly as the distance to the hyphal bridge increased (*p* < 0.001, Table 2), regardless of the AMF involved or the period of observation. However, a significant two-way interaction between distance and time was also observed (*p* < 0.001, Table 2). As the culturing time progressed, the relationship between distance and time shifted from strongly negative to positive (Figure 3B). Sporulation was predicted to occur preferentially near the hyphal bridge at 8–11 wpi, and farther away from the hyphal bridge at 12 wpi.

**FIGURE 4:**
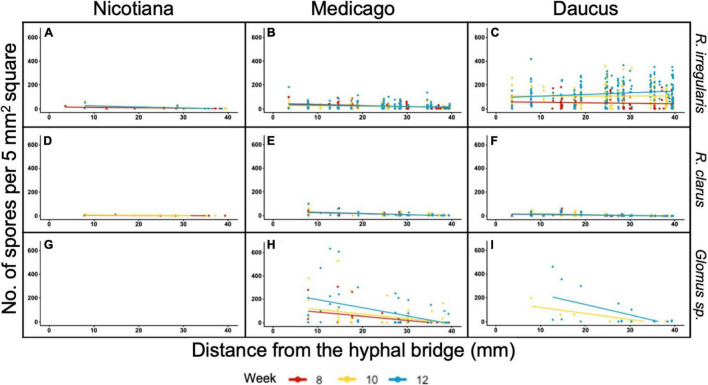
Distribution of spore counts scored in 5 mm^2^ grid squares in relation to their distance from the hyphal bridge. Regression lines were fit to spore counts recorded at 8 wpi (red), 10 wpi (yellow), and 12 wpi (blue). Panel columns are arranged by host (left to right: Nicotiana, Medicago, and Daucus) and rows are arranged by AMF (top to bottom: *R. irregularis, R. clarus*, and *Glomus* sp.).

We then assessed whether the significant effect of distance on sporulation was consistent amongst all pairs of host/AMF. The number of spores recorded in each square from 8 to 12 wpi was plotted against the distance variable for each combination of host/AMF (Figures 4A–I). Only the culture Daucus/*R. irregularis* exhibited a positive relationship between distance and spore count (Figure 4C). At 12 wpi, the spores were significantly more abundant at a farther distance from the hyphal bridge in the culture Daucus/*R. irregularis* compared to any of the other combinations of host/AMF. In contrast, the cultures Daucus/*Glomus* sp. and Medicago/*Glomus* sp. exhibited a negative relationship between distance and spore count (Figures 4H,I).

## Discussion

### *RocTest* as a generalizable tool for characterizing monoxenic cultures of arbuscular mycorrhizal fungi

*RocTest* is robust and standardized framework to assess the ROC performance in propagating AMF in monoxenic cultures. Monitoring the fungal propagation and sporulation for 12 weeks provided enough data for each culture to predict the propagation of AMF in symbiosis with different host roots. Specifically, the model predicted (1) the probability of the fungal culture progressing to any one of the six SD stages; and (2) the sporulation patterns in the HC. *RocTest* can be applied to assess the performance of any pair of host/AMF, and the profiles of stacked probability are independently comparable across laboratories, as long as the same experimental procedures are used. In addition, this method can easily be adapted to various *in vitro* systems, such as a one-compartment Petri dish or the autotrophic *in vitro* culture system developed by Voets et al. (2005). It can also be used to assess the impact of different culture media on AMF propagation. Finally, standardizing the assessment of ROC performance to support the propagation of AMF makes it feasible to build biobanks of well characterized Ri T-DNA transformed roots and to determine the best combination of host/AMF, which is an important asset for germplasm collections and AM fungal research.

### Collecting and modeling data for *RocTest*

The six stages of symbiosis development (SD) defined in this study capture the important phases of the fungal propagation process while considering the dynamic nature of the monoxenic culture: the period before symbiosis establishment (stages 0 to 2, before the first observed sporulation event), viability (stages 3 to 6, sporulation in the RC or HC), extraradical mycelium growth in the HC (stages 4 to 6), and sporulation in the HC (stage 6). More detailed observations would be time-consuming and reduce the sample size. Here, the sample size ranged from 8 to 68 cultures for any pair of host/AMF, at any time point. Because AM fungal growth is often variable, a large sample size is necessary to correct for any inconsistencies stemming from a smaller number of culture replicates. Among the six stages of SD, stage 2 (“roots and hyphae in close contact”) was the most subjective. A visual confirmation of hyphopodium would have been used to validate stage 2, but this was not possible to do under a stereomicroscope without destroying the culture. The quantification of spores in the HC incorporated aspects from various published methods developed for the purpose of making non-destructive, repeated observations of monoxenic AM cultures. For example, we used a 5 mm^2^ grid (Declerck et al., 1996) and randomized the observation areas (Koch et al., 2004; Ehinger et al., 2009). However, we also built upon previous studies by incorporating two key modifications. Since St-Arnaud et al. (1996) suggested that diffusible root exudates could inhibit the formation of spores of *R. irregularis* DAOM 197198/181602, spores were counted only in a root-free HC. Moreover, root-free compartments are not susceptible to obstructions by roots, which block spores from view and lead to underestimated spore counts. Spores were also counted in nine randomized, but evenly distributed areas in the HC. This reduced the potential for sampling bias and produced a sufficient amount of data to model the relative spatial distributions of HC sporulation for each combination of host/AMF.

The laser-cut grid-mount used to count spores in the HC prevents errors resulting from the inconsistent positioning of two-compartment Petri dishes and the 5 mm^2^ grid. For future studies, it would be beneficial to include hyphal measurements, as some fungal taxa in *Gigasporaceae* tend to prioritize hyphal growth over sporulation (De Souza et al., 2005). Hyphal measurements could complement spore counts obtained from each of the 5 mm^2^ areas selected, thus providing a more in-depth understanding of fungal behavior and phenotypic differences. Measurements could be taken by first imaging observation areas, and then using digital analysis tools such as NeuronJ or HyLength (Meijering et al., 2004; Cardini et al., 2020).

Finally, *RocTest* can be adapted to model the fungal propagation in a one-compartment Petri dish to accommodate AM species that produce spores close to the host roots and which do not propagate well in HC. In this case, the SD stages would be limited to stages 0 to 3, and the projector paper grid (in the grid-mount) would be modified to cover the entire surface of the Petri dish.

All the data collected for *RocTest* consist essentially of a time series in which each Petri dish was regularly observed to monitor the development and sporulation of the AMF. Mixed models were used to take into account the repeated nature of the sampling scheme (pseudo-replication) and the hierarchical structure of the data. For the SD assay, the probability of observing a culture in a given state at a given time could be estimated with a series of binomial model estimates. However, the multinomial cumulative link logit model was preferred, as it allowed the entire development to be modeled simultaneously throughout all stages. Moreover, this model made it possible to test for differences in the rate of fungal propagation in each combination of host/AMF. For modeling sporulation in the HC, a Poisson distribution was most appropriate, given that spore production is considered count data. Although it is possible for temporal autocorrelation to be present at the Petri dish level, the random selection of squares in each zone of the HC should reduce most of it. An autoregressive process for the residual was also added to the model of spore numbers, but this provided results that were qualitatively and quantitatively similar to the initial model.

### Host-specific effects are inconsistent across arbuscular mycorrhizal fungi

Modeling the SD and spore-count data with *RocTest* showed that the AMF associated with Medicago and Daucus exhibited divergent patterns of propagation, i.e., different rates of progression through the SD stages, variable spore production and distribution. In contrast, Nicotiana roots triggered a similar rate of progression through the SD stages, regardless of the AMF inoculated, but the propagation was mostly restricted to the RC. The Nicotiana/*R. clarus* pair was the least compatible, as only 83% of the culture replicates were likely to even germinate at 12 wpi. This lack of pre-symbiotic growth with *R. clarus* was specific to Nicotiana roots because the spores used to initiate the monoxenic cultures with each root species were sourced from the same culture. Overall, Nicotiana roots did not support viable monoxenic cultures, as the three species of AMF had a very low probability of completing their life cycle. In contrast, Zeng et al. (2018) observed similar root mycorrhization of *Medicago truncatula, Nicotiana benthamiana*, and *Allium shoeneprasum* by *R. irregularis* (DAOM 197198) under phosphorus-limited, *in vivo* conditions. Therefore, the poor compatibility between Nicotiana and AMF presented in this study may be attributed to either the *Rhizobium*-mediated transformation or the *in vitro* culturing conditions.

Unlike Nicotiana, the progression of AMF through the SD stages with either Medicago or Daucus roots differed depending on the identity of the fungus. Because *R. irregularis* exhibited a higher sporulation rate with Daucus relative to Medicago, it was expected that the fungus would progress faster through the SD stages with the former host. However, the profiles of stacked probabilities were very similar between the two types of monoxenic cultures. For *R. clarus*, the opposite trend was observed. The number of spores counted in the HC between each host species was not significantly different; however, the profiles of stacked probabilities were dissimilar between Daucus/*R. clarus* and Medicago/*R. clarus*. *Rhizophagus clarus* progressed faster through the SD stages when in symbiosis with Daucus than Medicago, as early as 4 wpi. When in symbiosis with Daucus, *R. clarus* developed an extensive hyphal network as opposed to producing spores (data not shown). This could explain the discrepancy between trends observed in SD and sporulation data.

The lack of consistency in the host-specific effects exhibited by Medicago and Daucus on the propagation of *R. irregularis* and *R. clarus* highlights that the identity of both symbionts is key in determining the symbiotic outcome. Host identity alone is insufficient to predict the growth response or morphology of a fungal partner (Cavagnaro et al., 2001; Koch et al., 2017). Recent studies have reported that the identities of both symbionts play a critical role in the resulting symbiosis through a genotype × genotype interaction (Mateus et al., 2019; Kokkoris et al., 2021). The *RocTest* analyses highlight the need to cross-compare new host root species with AMF, as novel hairy root species may be more compatible with recalcitrant AMF than the hairy roots currently available.

## Conclusion

Root organ cultures have been used to propagate arbuscular mycorrhizal fungi (AMF) for decades, but no standardized methodology has been available to assess their performance in the propagation of their fungal symbionts. *RocTest* offers an empirical means to assess the performance of hairy roots in propagating AMF in monoxenic cultures and to identify the best combination of host/AMF. This is achieved by plotting stacked probabilities inferred from the development of the fungal symbiont and analyzing the variability in propagation between two sets of host/AMF. *RocTest* also highlights phenotypic differences in sporulation. Germplasm collections and research on AMF require a greater diversity of transformed ROC in order to propagate a greater diversity of AMF under *in vitro* conditions. Through simple data collection and robust statistical modeling, *RocTest* provides the tools required to advance the development of *in vitro* cultures of AMF.

## Data availability statement

The datasets presented in this study can be found in online repositories. The names of the repository/repositories and accession number(s) can be found in the article/Supplementary material.

## Author contributions

DG, AM, and FS conceived and designed the experiments. CB provided the fungal inocula and the carrot ROC (P68). DG carried out all the experiments, wrote the first draft of the manuscript, and wrote the files in the OSF repository. FS rewrote the subsequent drafts. AM, JM, and FS supervised the data collection. JM developed the modeling method, supervised the statistical analyses, and wrote all of the sections related to modeling and statistics. JM and FS revised the R scripts. All authors contributed critically to the drafts and gave final approval for publication.

## Footnotes

1
http://amf-phylogeny.com/

2
https://bccm.belspo.be

3
https://agriculture.canada.ca/en/agricultural-science-and-innovation/agriculture-and-agri-food-research-centres-and-collections/canadian-collection-arbuscular-mycorrhizal-fungi-ccamf

4
https://doi.org/10.17605/OSF.IO/2NKU8
