# Supplementary material for: RocTest: A standardized method to assess the performance of root organ cultures in the propagation of arbuscular mycorrhizal fungi
Source: Front Microbiol. 2022 Jul 28;13:937912. doi: 10.3389/fmicb.2022.937912 (PMC9366734; doi:10.3389/fmicb.2022.937912)
Supplement: Supplementary file 1 [file Data_Sheet_1.docx]

**Supporting information**

*Hairy root transformation*

Three hairy root hosts were used in this study, generated from *Nicotiana benthamiana* (hereafter Nicotiana), *Medicago truncatula* (ecotype R108; hereafter Medicago), and *D. carota* (clone P68; hereafter Daucus). Daucus ROC were obtained from CCAMF, while Nicotiana and Medicago ROC were generated from seeds provided by the Boyce Thompson Institute, Cornell University, Ithaca, NY, USA.

Transgenic Medicago roots were generated according to the methods described by Floss *et al.* (2013) up to section 3.2.3 ‘Induction of Transgenic Roots’, step 8, where the following modifications were made: hairy roots were excised and aseptically transferred to a M medium modified for the Medicago Root Culture (hereafter MRC medium, described page 3) and containing 200 µg/ml of cefotaxime to eliminate any residual *Rhizobium rhizogenes*. Hairy roots were cultured upon MRC media for six weeks, sub-culturing onto fresh media every two weeks to ensure cultures were axenic. Next, Medicago hairy roots were transferred to M-Media (Bécard & Fortin, 1988) modified by adding 20 g/l of sucrose and increasing the pH to 6.0, gelled with 0.3% phytagel (Sigma-Aldrich).

Transgenic Nicotiana roots were generated by first sowing seeds on F medium (Floss *et al.*, 2013) in a squared Petri dish and allowed to germinate at room temperature on a windowsill under natural light until seedling roots were approximately a centimetre in length. The day before seedling transformation, ARqua1 was added to Luria-Bertanbi (LB) and incubated overnight at 28ºC, shaking at 200 rpm. The following day, the overnight culture was used to initiate secondary ARqua1 cultures in 50 ml Falcon tubes and incubated at 28ºC (shaking at 200 rpm) until an optical density_600nm_ reading between 0.5–1 was recorded. Next, the ARqua1 cells were harvested by centrifuging at a speed of 2,900 g at 28ºC for 10 min. A bacterial suspension was then created by re-suspending the ARqua1 pellet in MS medium (Murashige & Skoog, 1962), and then transferred to one-compartment Petri dishes. To increase *Agrobacterium* virulence, 100 µmol of acetosyringone was added to the ARqua1 suspension. Under a flow hood, Nicotiana root tips were removed from seedlings before they were placed into the ARqua1 suspension and oriented such that sectioned roots were entirely submerged. Seedlings were left in the suspension for an hour before they were transferred to square Petri dishes, where their roots were carefully pressed into F medium mixed with kanamycin (50 µg/ml). Incubation of Nicotiana seedlings was then performed according to steps 7 and 8 under section 3.2.3 ‘Induction of Transgenic Roots’ in Floss *et al.* (2013).

*Root transformation efficiency and maintenance*

The Ri T-DNA transformation efficiency differed between Medicago and Nicotiana, with at least one hairy root per seedling for the former, but only one hairy root per 14 seedlings for the latter. Despite this difference, the hairy roots from both plant species were propagated quickly and consistently through routine subculture. In two-compartment Petri dishes, maintenance consisted of re-directing and trimming roots growing over the barrier between compartments to ensure that the HC would remain root-free. Despite working aseptically, the cultures that required the most maintenance had the highest risk of being contaminated. Medicago cultures had the highest likelihood of remaining monoxenic, as they required the least maintenance overall (Supplementary Table **S2**). The other two host species were found to possess higher likelihoods of encountering airborne contaminants due to the presence of numerous root hairs for Nicotiana or the excess production of condensation within Petri dishes for Daucus (Supplementary Fig. **S1**).

***Recording the data required to run RocTest***

Identifying and recording the SD stages was quick and simple: bi-weekly observations under a stereomicroscope took less than 5 minutes per culture (Fig. **1**), and data were recorded in a simple table (‘SD_data.csv’ on OSF). Counting spores took a longer time and varied depending on the host and AM fungal pair, as well as the period of observation. For example, at 12 wpi, the mean number of spores per square (n = 27) was 10 ± 4 and 126 ± 6 for the pairs Medicago / *R. clarus*, and Daucus / *R. irregularis*, respectively (Supplementary Fig. **S2**).

**MRC (Medicago Root Culture) medium recipe.** There are four stock solutions to prepare: N6 major salts, SH minor salts, SH vitamins, and EDFS.

**N6 Major Salts** (store at 4°C after autoclaving)

Chemical Gram per litre

MgSO_4_x7H_2_O 1.85

KNO_3_ 1.415

(NH_4_)_2_SO_4_ 4.63

CaCl_2_x2H_2_O 1.66

KH_2_PO_4_  0.1

**SH Minor Salts** (store at 4°C without autoclaving)

Chemical Milligram per 100 ml

MnSO_4_xH_2_O 1000

H_3_BO_3_ 500

ZnSO_4_ x 7H_2_O 100

KI 100

Na_2_MoO_4_x2H_2_O 10

CuSO_4_ 12.8

CoCl_2_x6H_2_O 10

**SH Vitamins** (store at 4°C without autoclaving)

Chemical Milligram per 100 ml

Nicotinic acid 500

Thiamine HCl (B1 vit) 500

Pyridoxine HCl (B6 vit) 500

**EDFS stock solution** (store at 4°C without autoclaving)

EDFS (Ferric-EDTA) 697.4 mg per 100 ml

**Mixture of stock solutions to prepare 1 l of MRC medium:**

Chemicals or Stock Per 1 l

N6 Major Salts 50 ml

SH Minor Salts 0.5 ml

SH Vitamins 0.5 ml

EDFS Stock 10 ml

Myo-Inositol 50 mg

Sucrose 10 g

**pH 5.8**

Kalys Agar 5.0 g

**Table S1.** List of species of AM fungi, strain DAOMC numbers, and origin.

| **Species** | **Strain / DAOMC no.** | **Origin** |
| --- | --- | --- |
| *Rhizophagus irregularis* | 4375 / DAOMC234181 | Magdalens Islands, QC |
| *Rhizophagus clarus* | GC-5 / DAOMC234281 | La Palma, Cuba |
| *Glomus* sp. | GC-4 / DAOMC240160 | Angers, France |

**Table S2.** Number of monoxenic cultures set for each pair of host / AM fungal species and percentage of cultures free of contamination for each host species during the 12 weeks of incubation.

| **Host** | **Week** | ***R. irregularis*** | ***R. clarus*** | ***Glomus* sp.** | **Total** | **Percentage of cultures free of contamination (%)** |
| --- | --- | --- | --- | --- | --- | --- |
| **Nicotiana** | 2 | 54 | 45 | 40 | 139 | 100 |
|  | 4 | 37 | 32 | 40 | 109 | 78.4 |
|  | 6 | 34 | 25 | 34 | 93 | 66.9 |
|  | 8 | 22 | 20 | 32 | 74 | 53.2 |
|  | 10 | 18 | 20 | 30 | 68 | 48.9 |
|  | 12 | 8 | 13 | 21 | 42 | 30.2 |
| **Medicago** | 2 | 64 | 45 | 46 | 155 | 100 |
|  | 4 | 60 | 38 | 45 | 143 | 92.2 |
|  | 6 | 50 | 34 | 43 | 127 | 81.9 |
|  | 8 | 30 | 33 | 41 | 104 | 67.1 |
|  | 10 | 28 | 33 | 37 | 98 | 63.2 |
|  | 12 | 16 | 23 | 27 | 66 | 42.6 |
| **Daucus** | 2 | 68 | 40 | 37 | 145 | 100 |
|  | 4 | 67 | 37 | 36 | 140 | 96.6 |
|  | 6 | 58 | 37 | 34 | 129 | 89 |
|  | 8 | 22 | 32 | 34 | 88 | 60.7 |
|  | 10 | 21 | 29 | 29 | 79 | 54.5 |
|  | 12 | 19 | 21 | 15 | 55 | 37.9 |

**Table S3.** Sample size for each pair of host / AMF. ‘No. of cultures’ refers to the number of two-compartment Petri dishes that remained monoxenic at 8-, 10-, and 12-weeks post inoculation. ‘No. of observations’ refers to the nine 5 mm^2^ squares randomly distributed in the HC of each culture.

| **AM fungal species** | Week | **Nicotiana** | | **Medicago** | | **Daucus** | |
| --- | --- | --- | --- | --- | --- | --- | --- |
|  |  | No. of cultures | No. of observations | No. of cultures | No. of observations | No. of cultures | No. of observations |
| *R. irregularis* | 8 | 1 | 9 | 10 | 90 | 14 | 126 |
|  | 10 | 1 | 9 | 16 | 144 | 21 | 189 |
|  | 12 | 1 | 9 | 21 | 189 | 26 | 234 |
| *R. clarus* | 8 | 1 | 9 | 4 | 36 | 7 | 63 |
|  | 10 | 1 | 9 | 5 | 45 | 11 | 99 |
|  | 12 | 0 | 0 | 3 | 27 | 8 | 72 |
| *Glomus* sp. | 8 | 0 | 0 | 4 | 36 | 0 | 0 |
|  | 10 | 0 | 0 | 6 | 54 | 1 | 9 |
|  | 12 | 0 | 0 | 6 | 54 | 3 | 27 |

**Table S4.** Post hoc pairwise comparisons between the pairs of host / AMF used in the linear mixed model. Estimates, standard error (SE), degrees of freedom (Df), and *p* values were obtained from Tukey HSD tests. Estimate is the difference in number of spores per square predicted at week 10.

| Pairwise comparison | | Estimate | SE | Df | *p* value |
| --- | --- | --- | --- | --- | --- |
| Pair 1 | Pair 2 |  |  |  |  |
| Nic / *R. clarus* | Nic / *R. irregularis* | -1.24 | 1.99 | Inf | 0.99 |
| Nic / *R. clarus* | Dau / *R. clarus* | -0.39 | 1.47 | Inf | 0.99 |
| Nic / *R. clarus* | Dau / *R. irregularis* | -3.21 | 1.44 | Inf | 0.22 |
| Nic / *R. clarus* | Med / *R. clarus* | -0.82 | 1.55 | Inf | 0.99 |
| Nic / *R. clarus* | Med / *R. irregularis* | -1.51 | 1.45 | Inf | 0.90 |
| Nic / *R. irregularis* | Dau / *R. clarus* | 0.85 | 1.44 | Inf | 0.99 |
| Nic / *R. irregularis* | Dau / *R. irregularis* | -1.98 | 1.41 | Inf | 0.73 |
| Nic / *R. irregularis* | Med / *R. clarus* | 0.42 | 1.52 | Inf | 0.99 |
| Nic / *R. irregularis* | Med / *R. irregularis* | -0.27 | 1.42 | Inf | 1.00 |
| Dau / *R. clarus* | Dau / *R. irregularis* | -2.83 | 0.45 | Inf | **< 1.0e-4^*^** |
| Dau / *R. clarus* | Med / *R. clarus* | -0.44 | 0.73 | Inf | 0.99 |
| Dau / *R. clarus* | Med / *R. irregularis* | -1.13 | 0.48 | Inf | 0.17 |
| Dau / *R. irregularis* | Med / *R. clarus* | 2.39 | 0.68 | Inf | **5.5e-3^*^** |
| Dau / *R. irregularis* | Med / *R. irregularis* | 1.70 | 0.39 | Inf | **2.0e-4^*^** |
| Dau / *R. clarus* | Med / *R. irregularis* | -0.69 | 0.69 | Inf | 0.92 |

Inf: infinite degrees of freedom

***
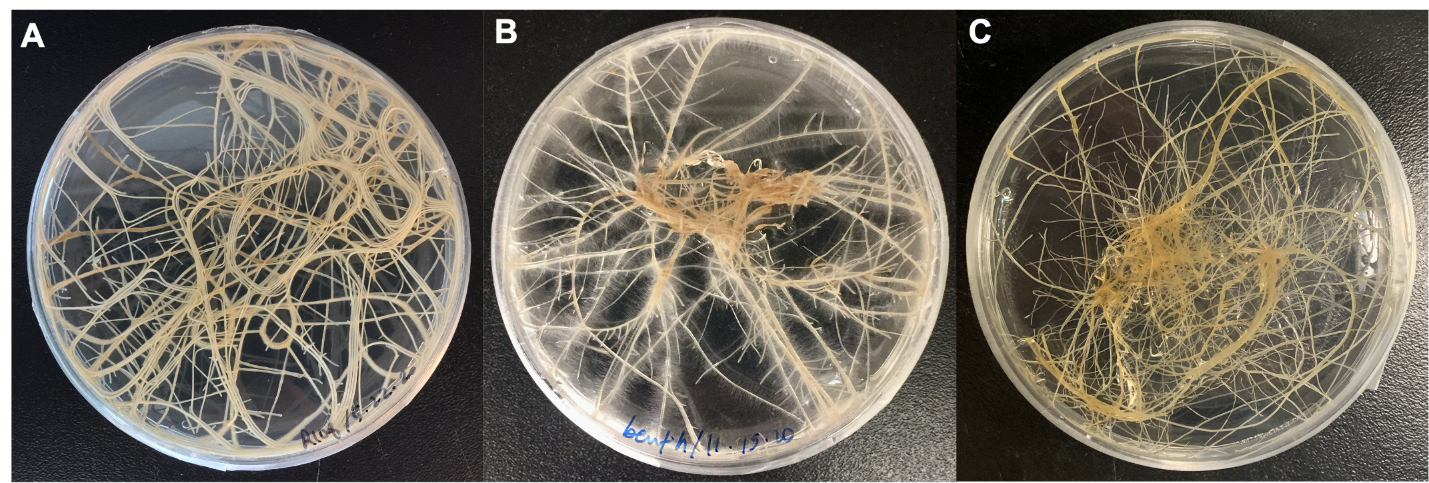
*Fig. S1.** Ri T-DNA transformed roots of (A) Medicago (cv. R108)*,* (B) Nicotiana*,* and (C) Daucus (strain P68).


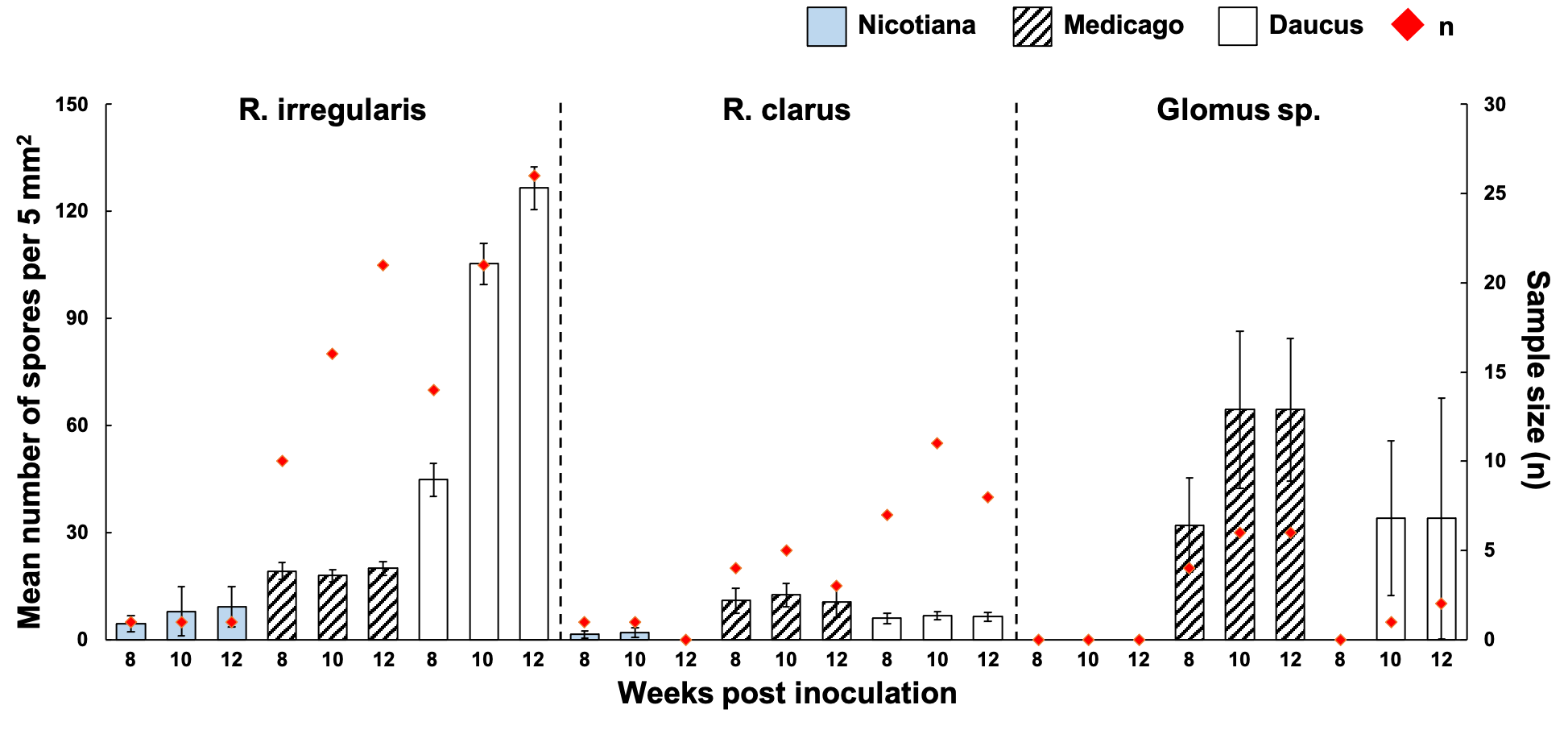
**Fig. S2.** Number of spores per 5mm^2^ (Mean ± SE) in HC of each host (Nicotiana- blue bars, Medicago – dashed pattern bars, and Daucus – white bars) and AMF pair from 8-12 wpi. Sample size (n) plotted on a secondary axis (on right) denoted by a red diamond.

**References**

Bécard G, Fortin JA. 1988. Early events of vesicular–arbuscular mycorrhiza formation on Ri T‐DNA transformed roots. *New Phytologist* 108: 211–218.

Floss DS, Schmitz AM, Starker CG, Gantt JS, Harrison MJ. 2013. Gene Silencing in *Medicago truncatula* Roots Using RNAi. In: Rose RJ, ed. *Methods in Molecular Biology*. 163–177.

Murashige T, Skoog F. 1962. A Revised Medium for Rapid Growth and Bio Assays with Tobacco Tissue Cultures. *Physiologia Plantarum* 15: 473–497.
